# Left Atrial Myxoma Surgery in Cryoglobulinemic Vasculitis Associated with Hepatitis B: A Clinical Case Report

**DOI:** 10.3390/reports9020101

**Published:** 2026-03-27

**Authors:** Iustina Maria Andrieș, Radu Sebastian Gavril, Cristina Andreea Adam, Grigore Tinica, Florin Mitu

**Affiliations:** 1 Grigore T. Popa University of Medicineand Pharmacy, 700115 Iasi, Romania; andries.iustina@yahoo.com (I.M.A.); cristina-andreea_adam@umfiasi.ro (C.A.A.); grigore.tinica@umfiasi.ro (G.T.); florin.mitu@umfiasi.ro (F.M.); 2 Romanian Academy of Medical Sciences, 927180 Bucharest, Romania; 3 Romanian Academy of Scientists, 050044 Bucharest, Romania

**Keywords:** left atrial myxoma, cardiac rehabilitation, postoperative atrial fibrillation, palpable purpura, cryoglobulinemic vasculitis, hepatitis B virus

## Abstract

**Background and Clinical Significance**: Left atrial myxoma is the most common benign primary cardiac tumor and is associated with embolic and hemodynamic complications. Complete surgical excision is the treatment of choice, while postoperative cardiovascular rehabilitation is essential for functional recovery. **Case Presentation**: We report the case of a 75-year-old woman with arterial hypertension, dyslipidemia, and chronic venous insufficiency (Clinical–Etiological–Anatomical–Pathophysiological (CEAP) class 2), and chronic hepatitis B virus (HBV) infection who underwent surgical excision of a left atrial myxoma and was subsequently admitted three weeks postoperatively for phase II cardiovascular rehabilitation. The postoperative course was complicated by transient atrial fibrillation, peripheral edema, pleural effusion, and progressive purpuric lesions of the lower limbs. Laboratory and immunological evaluation revealed positive cryoglobulins, markedly elevated rheumatoid factor (1058 UI/mL) and IgM levels (715 mg/dL), reduced complement levels (C3, C4), normocytic normochromic anemia, microscopic hematuria, and elevated ALT (156 U/L), AST (142 U/L), total bilirubin (1.4 mg/dL), and INR (1.6), suggestive of hepatic inflammatory activity. HBV status was scheduled for evaluation through Gastroenterology referral (HBV DNA viral load, serological markers: HBsAg, HBeAg, anti-HBe), as our Cardiology Rehabilitation Clinic lacks the possibility of evaluation. After systematic exclusion of alternative etiologies, secondary cryoglobulinemic vasculitis in the context of chronic HBV infection with biochemical evidence of hepatic activity was considered the most plausible diagnosis. **Conclusions**: This case highlights the complexity of managing elderly patients after cardiac tumor surgery, particularly in the presence of systemic comorbidities. Early recognition of extracardiac complications and an individualized, multidisciplinary strategy are essential to optimize outcomes.

## 1. Introduction and Clinical Significance

Left atrial myxoma is the most frequent primary cardiac tumor and may present with obstructive symptoms, embolic phenomena, and systemic manifestations such as fever, weight loss, and fatigue [1,2,3]. These systemic features have been linked to inflammatory cytokine release and may occasionally mimic autoimmune or vasculitic syndromes, complicating diagnostic reasoning and postoperative monitoring [1,2].

Complete surgical excision is generally curative; however, the postoperative period may be complicated by arrhythmias, transient heart failure, pleural effusion, and extracardiac manifestations related to comorbid conditions or systemic inflammation [3,4,5]. Cardiovascular rehabilitation plays a central role in restoring functional capacity and quality of life after cardiac surgery.

HBV-related cryoglobulinemic vasculitis is a recognized extrahepatic manifestation of chronic HBV infection, although it is less common than HCV-associated disease [6,7]. It may present with palpable purpura, renal abnormalities, and systemic inflammatory features and often requires coordinated antiviral and immunologic evaluation [6,7]. We report a case illustrating the diagnostic and management challenges posed by suspected HBV-related cryoglobulinemic vasculitis developing in the early postoperative rehabilitation period following left atrial myxoma resection.

## 2. Case Presentation

**Figure 1:**
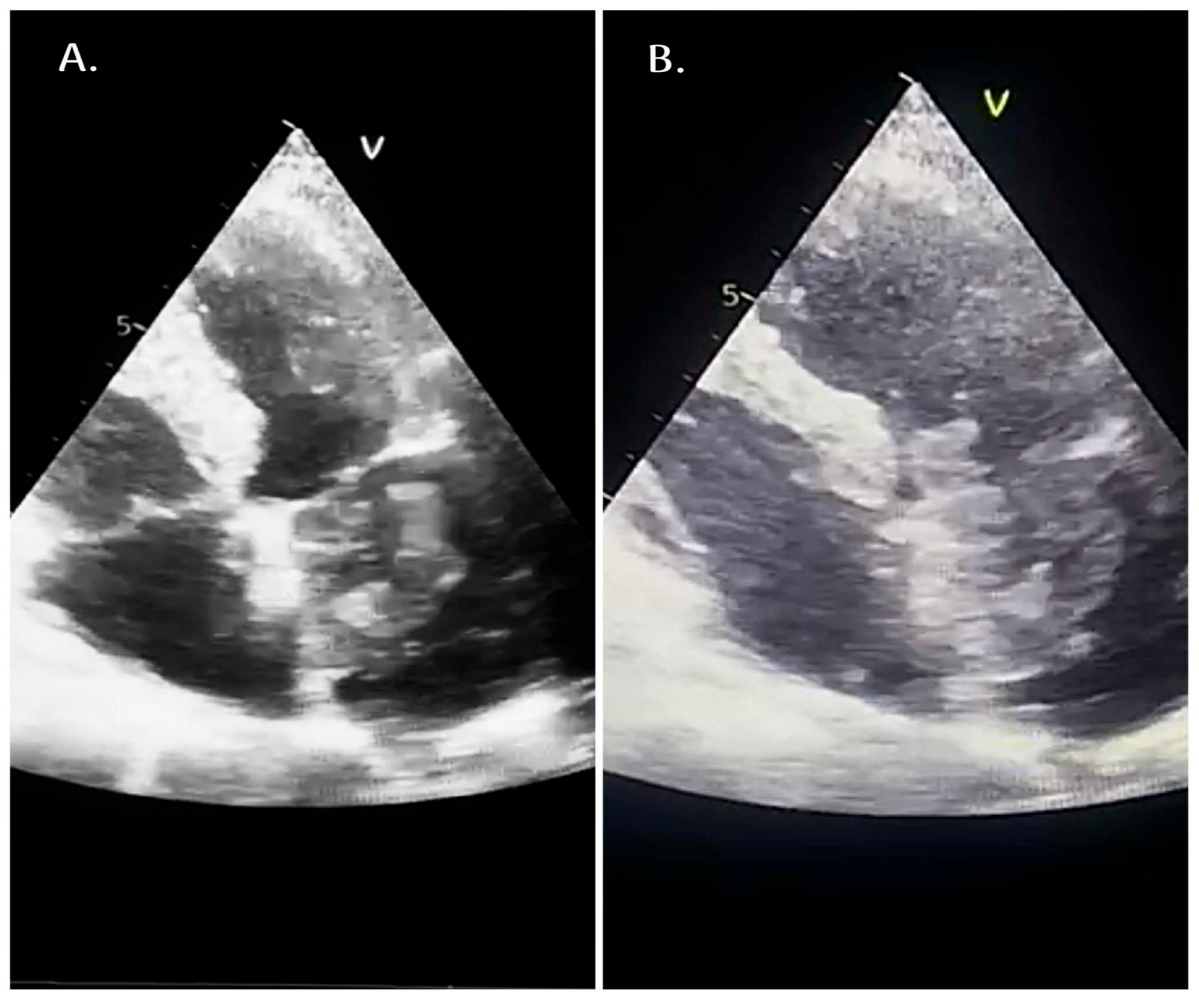
(**A**) Apical four-chamber transthoracic echocardiographic view showing a heterogeneous intracavitary mass within the left atrium, suggestive of atrial myxoma. (**B**) A different cardiac cycle frame illustrating the mobility of the left atrial mass.

A 75-year-old woman with essential arterial hypertension (very high cardiovascular risk), dyslipidemia, and chronic venous insufficiency (Clinical–Etiological–Anatomical–Pathophysiological (CEAP) class 2) was diagnosed four months prior with a left atrial mass. Transthoracic echocardiography demonstrated a heterogeneous, mobile intracavitary mass within the left atrium consistent with atrial myxoma (Figure 1A,B). She underwent surgical excision. The immediate postoperative course was complicated by transient atrial fibrillation, successfully managed with pharmacological therapy.

The patient was admitted three weeks after surgery to a cardiovascular rehabilitation clinic for Phase II rehabilitation. On admission, she was classified as NYHA class II. A 6 min walk test demonstrated reduced functional capacity. A structured supervised rehabilitation program was initiated, and progressive improvement in exercise tolerance and walking distance was documented during hospitalization. On admission, vital signs were stable (blood pressure 130/70 mmHg, heart rate 72 bpm). Physical examination revealed diminished breath sounds at the right lung base, mild bilateral lower limb edema, and purpuric lesions on the lower extremities.

Comprehensive laboratory testing revealed normocytic normochromic anemia (hemoglobin decreased from 12.3 g/dL to 9.0 g/dL during hospitalization), elevated erythrocyte sedimentation rate, increased C-reactive protein (5.37 mg/dL), markedly elevated rheumatoid factor (1058 UI/mL), increased IgM levels (715 mg/dL), reduced IgG levels (437 mg/dL), reduced complement levels (C3 and C4), and positive cryoglobulins. Urinalysis showed persistent microscopic hematuria with hyaline casts. Liver function tests demonstrated elevated ALT (156 U/L), AST (142 U/L), total bilirubin (1.4 mg/dL), and INR (1.6), suggestive of hepatic inflammatory activity. Given palpable purpura with a normal platelet count, an immunologic workup was initiated. Rheumatoid factor was elevated with normal anti-cyclic citrullinated peptide antibodies, and IgM levels were increased. Peripheral smear and abdominal ultrasound did not reveal malignant or organ-specific pathology; however, cryoglobulins were positive, raising suspicion of cryoglobulinemic vasculitis. HBV infection had been recently diagnosed, supporting the working hypothesis of secondary cryoglobulinemic vasculitis in the context of chronic HBV infection. HBV status was scheduled for evaluation through Gastroenterology referral (HBV DNA viral load, serological markers: HBsAg, HBeAg, anti-HBe), as our Cardiology Rehabilitation Clinic lacks the possibility of evaluation.

**Figure 2:**
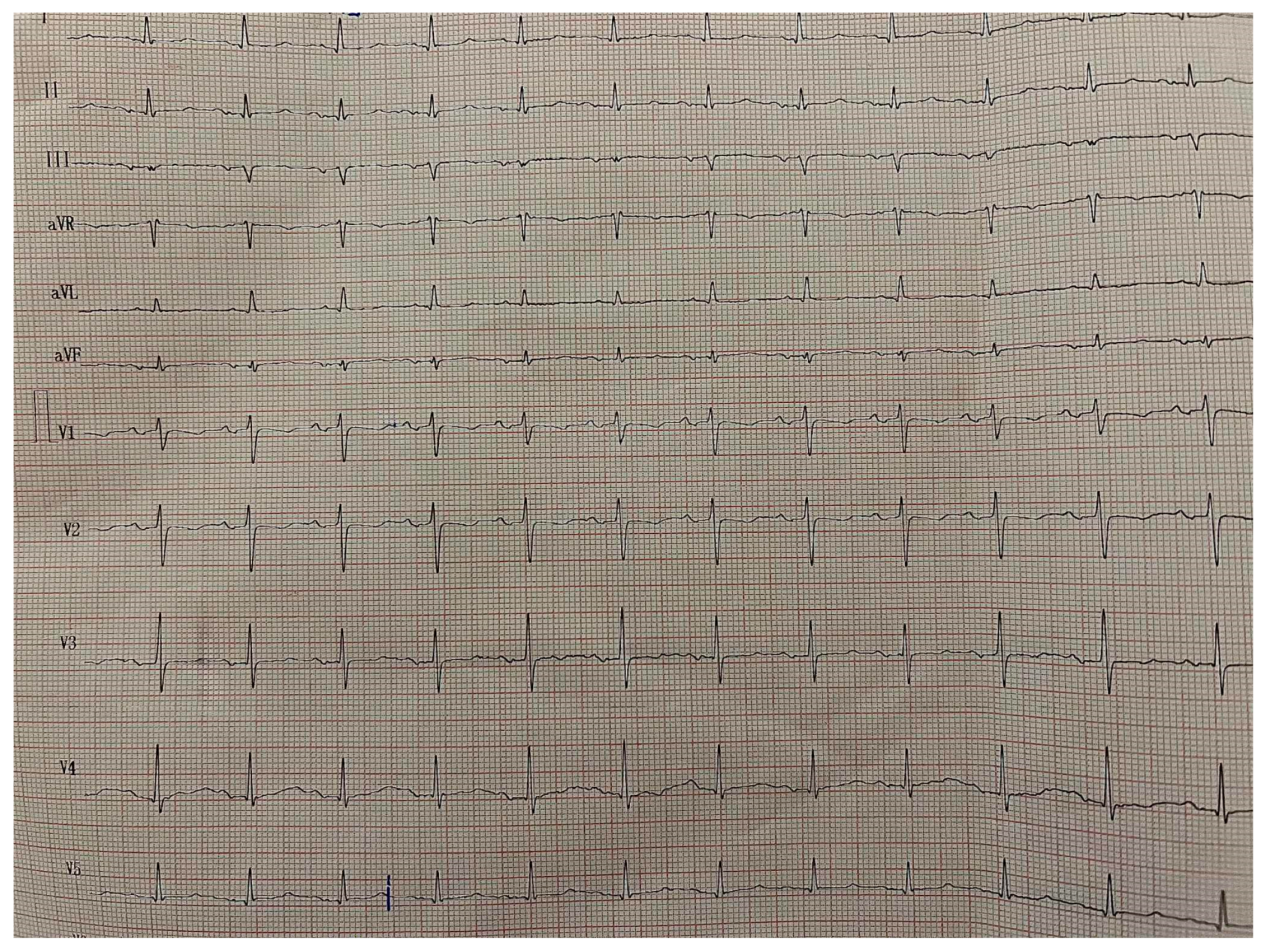
Twelve-lead electrocardiogram showing sinus rhythm without ischemic changes during hospitalization.

A 12-lead electrocardiogram during hospitalization showed sinus rhythm with normal atrioventricular conduction and no ischemic changes (Figure 2).

**Figure 3:**
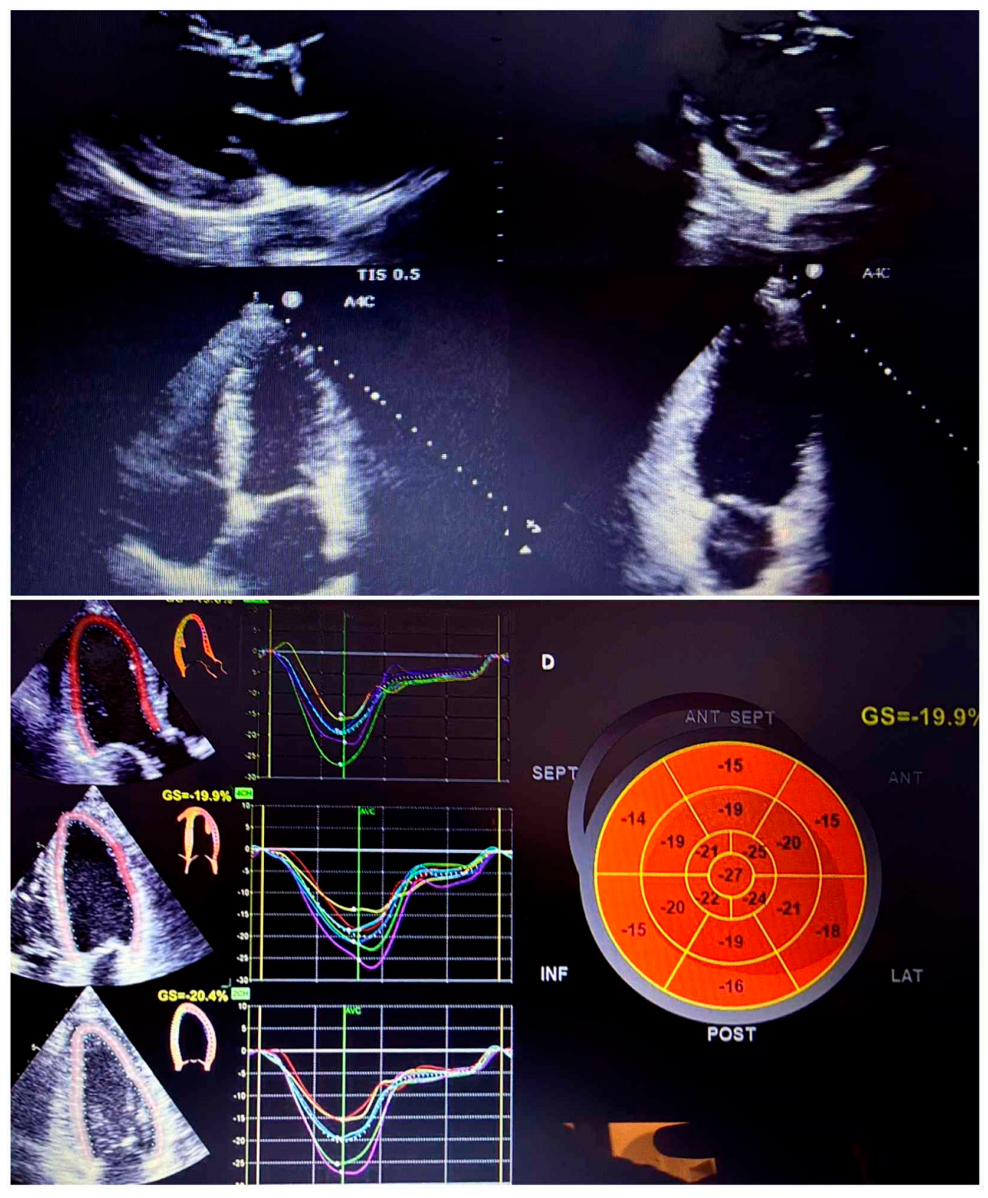
Transthoracic echocardiography showing preserved systolic function and normal global longitudinal strain (−19.9%), associated with aortic atheromatosis and posterior mitral annular calcification.

Transthoracic echocardiography demonstrated concentric left ventricular hypertrophy with preserved systolic function and a global longitudinal strain of −19.9%, together with aortic atheromatosis and posterior mitral annular calcification (Figure 3).

Holter ECG monitoring revealed persistent sinus rhythm without pauses or malignant arrhythmias.

**Figure 4:**
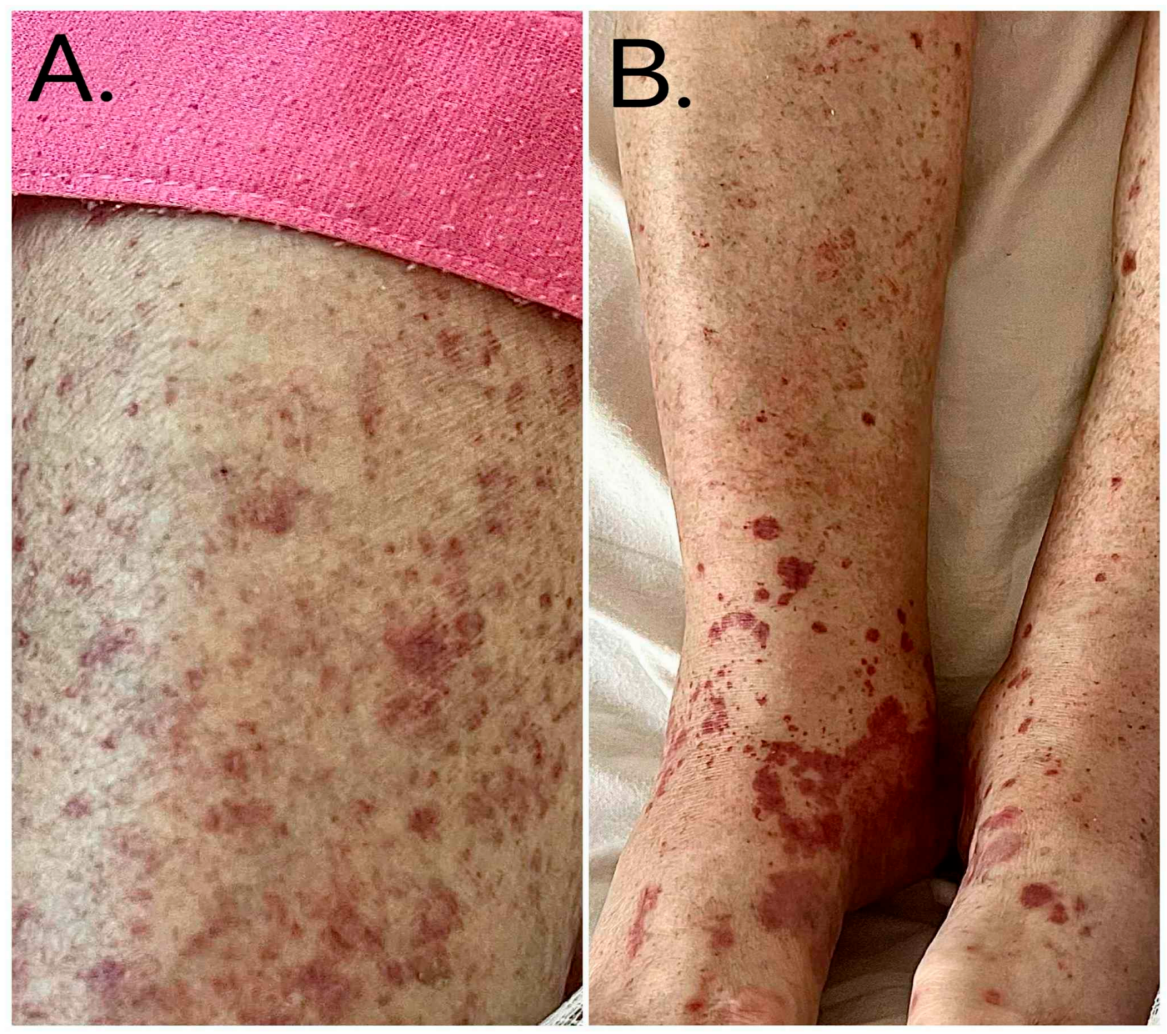
(**A**) Diffuse bilateral purpuric rash involving the lower extremities observed during hospitalization. (**B**). Palpable purpuric lesions of the lower limbs with distal predominance, suggestive of small-vessel vasculitis.

During hospitalization, the patient developed worsening lower limb edema and progressive palpable purpura with distal predominance, consistent with a small-vessel vasculitic process (Figure 4). She also developed dyspnea; imaging confirmed a moderate right-sided pleural effusion. Calcium channel blockers were discontinued, and intravenous furosemide was initiated, leading to improvement in dyspnea and edema.

Three days prior to evaluation, the patient reported an episode of rectorrhagia. In the context of anemia and recent bleeding, apixaban was temporarily withheld and later resumed at a reduced dose at discharge, considering the history of postoperative atrial fibrillation and persistent anemia.

Given the progression of purpuric lesions, additional hematologic and rheumatologic evaluation was conducted to clarify the differential diagnosis of postoperative purpura. Alternative etiologies considered included anticoagulation-related cutaneous lesions, thrombocytopenia (including heparin-induced thrombocytopenia), septic emboli, cholesterol embolization syndrome, and hypersensitivity vasculitis. These possibilities were systematically evaluated and excluded based on normal platelet count, coagulation profile, absence of infectious or embolic evidence, and compatible immunological findings (Table 1).

Considering chronic HBV infection with biochemical evidence of hepatic inflammatory activity and positive cryoglobulins, secondary cryoglobulinemic vasculitis was considered the most plausible diagnosis. Gastroenterology referral was recommended for evaluation of viral replication and initiation of antiviral therapy. Skin biopsy was not performed due to institutional limitations, representing a limitation of this report.

## 3. Discussion

This case illustrates the complexity of postoperative management in an elderly patient following left atrial myxoma excision. While surgical treatment is typically curative, postoperative complications—particularly arrhythmias and heart failure-related congestion—may occur and can affect rehabilitation progress [3,4,5]. Cardiac surgery is associated with a systemic inflammatory response mediated by cytokine release, complement activation, and endothelial dysfunction, particularly in the setting of cardiopulmonary bypass. Transient postoperative hemodynamic changes may further amplify inflammatory phenomena. Although atrial fibrillation and peripheral edema are common postoperative findings, no direct evidence supported a primary etiological role in the vasculitic process. However, the postoperative inflammatory milieu may have acted as a facilitating factor.

Cryoglobulinemic vasculitis is characterized by immune-complex deposition and typically presents with palpable purpura, arthralgias, weakness, and renal abnormalities. Although most commonly associated with hepatitis C, HBV-related cryoglobulinemic vasculitis is a recognized entity and should be considered when cryoglobulins are detected in a patient with an active HBV infection [6,7].

Another layer of complexity in this case was anticoagulation management in the context of postoperative atrial fibrillation, anemia, and recent bleeding. The need for temporary interruption and dose-adjusted resumption of anticoagulation emphasized the importance of individualized risk–benefit assessment.

It is also notable that myxoma itself may be associated with systemic inflammatory manifestations via cytokine release, potentially confounding the differentiation between postoperative inflammation and true systemic vasculitis [1,2]. In this context, the concurrence of positive cryoglobulins, purpura progression, urinary abnormalities, and chronic HBV infection with biochemical evidence of hepatic inflammatory activity supported the working diagnosis of secondary cryoglobulinemic vasculitis as the most coherent explanation; however, in the absence of HBV DNA quantification and histopathological confirmation, a definitive causal relationship cannot be established. This underscores the importance of multidisciplinary assessment—including cardiology, rehabilitation, gastroenterology, and rheumatology—in complex postoperative patients.

## 4. Conclusions

Postoperative cardiovascular rehabilitation is an essential component of recovery after left atrial myxoma surgery, improving functional capacity and overall prognosis. In elderly patients with systemic comorbidities, early recognition of extracardiac complications is crucial. In this case, suspected HBV-related cryoglobulinemic vasculitis significantly influenced clinical decision-making, including diuretic strategy, anticoagulant adjustment, and the need for specialist referral and antiviral therapy. A personalized multidisciplinary approach and close follow-up are fundamental to optimize outcomes.

## Figures and Tables

**Table 1:** Laboratory tests at admission and during hospitalization.

Parameter	At Admission	During Hospitalization	Reference Range
Hemoglobin (g/dL)	12.3	9.0	12.0–16.0
ESR (mm/h)	51	Elevated	<20
CRP (mg/dL)	0.01	5.37	<0.5
Fibrinogen (mg/dL)	446	—	200–400
Lymphocytes (/mm^3^)	800	Decreased	1000–4000
Rheumatoid factor (UI/mL)	—	1058	<14
IgM (mg/dL)	—	715	40–230
IgG (mg/dL)	—	437	700–1600
Cryoglobulins	—	Positive	negative
Urinalysis	hematuria, casts	Persistent	negative
Complement C3	—	Decreased	normal
Complement C4	—	Decreased	normal
ALT (U/L)	38	156	7–35
AST (U/L)	34	142	8–35
Total Bilirubin (mg/dL)	1.1	1.4	0.2–1.2
INR	1.1	1.6	0.8–1.2

Abbreviations: ESR—erythrocyte sedimentation rate; CRP—C-reactive protein; Ig—immunoglobulin; ALT—alanine aminotransferase; AST—aspartate aminotransferase; INR—international normalized ratio.

## Data Availability

The original contributions presented in this study are included in the article. Further inquiries can be directed to the corresponding author.
